# Deep learning in holography and coherent imaging

**DOI:** 10.1038/s41377-019-0196-0

**Published:** 2019-09-11

**Authors:** Yair Rivenson, Yichen Wu, Aydogan Ozcan

**Affiliations:** 1 Electrical and Computer Engineering Department, University of California, Los Angeles, CA 90095 USA; 2 Bioengineering Department, University of California, Los Angeles, CA 90095 USA; 3 California NanoSystems Institute (CNSI), University of California, Los Angeles, CA 90095 USA; 4 Department of Surgery, David Geffen School of Medicine, University of California, Los Angeles, CA 90095 USA

**Keywords:** Imaging and sensing, Phase-contrast microscopy, Microscopy

## Abstract

Recent advances in deep learning have given rise to a new paradigm of holographic image reconstruction and phase recovery techniques with real-time performance. Through data-driven approaches, these emerging techniques have overcome some of the challenges associated with existing holographic image reconstruction methods while also minimizing the hardware requirements of holography. These recent advances open up a myriad of new opportunities for the use of coherent imaging systems in biomedical and engineering research and related applications.

## Imaging: Deep learning benefits

Deep learning enabled by neural networks is bringing a host of new opportunities for improving the reconstruction of images obtained from digital holography and coherent imaging schemes. In a discussion of the topic, Yair Rivenson, Yichen Wu, and Aydogan Ozcan from the University of California at Los Angeles, USA explain how once “trained” with appropriate datasets, neural networks can learn to reconstruct images with added benefits such as improved phase recovery and extended depth of field as well as enhanced spatial resolution and superior signal-to-noise ratio. Advances in computing power, especially graphics processing units (GPUs), mean that such image reconstructions can now typically take place in a fraction of a second on a low-cost platform. The advances are expected to impact various applications in e.g., biomedical imaging, environmental sensing and materials science.

## Introduction

Exponential advancements in computational resources and algorithms have given rise to new paradigms in microscopic imaging modalities that rely on computation to digitally reconstruct and enhance images, surpassing the capabilities of conventional microscopes. Among these computational microscopy modalities, digital holographic microscopy (DHM) provides several unique opportunities by encoding a complex 3D optical field into intensity modulations through the interference of scattered sample waves and a reference wave, which forms a hologram of the sample. Some important advantages of holography include label-free imaging of samples at a low-radiation dose, inference of the objects’ phase distribution (especially useful for the imaging of, e.g., live cells and other biological specimens within a liquid environment)^1,2^, and numerical 3D refocusing throughout the sample volume by processing a single hologram, i.e., without any mechanical scanning. Despite these important advantages, digital holographic microscopy systems are not as widely used as other microscopy modalities, such as brightfield or fluorescence microscopes. The wide applicability of digital holographic microscopes is partially bottlenecked by some challenges: the “missing phase problem” in holography requires phase recovery, which is often implemented using iterative methods that demand the acquisition of additional measurements using relatively complex and alignment-sensitive imaging set-ups; furthermore, even after the phase recovery step, coherence-related artifacts appear in the reconstructed images in the form of, e.g., speckle noise and multiple-reflection interference, which altogether degrade the image contrast compared to, e.g., brightfield or fluorescence microscopy.

**Fig. 1:**
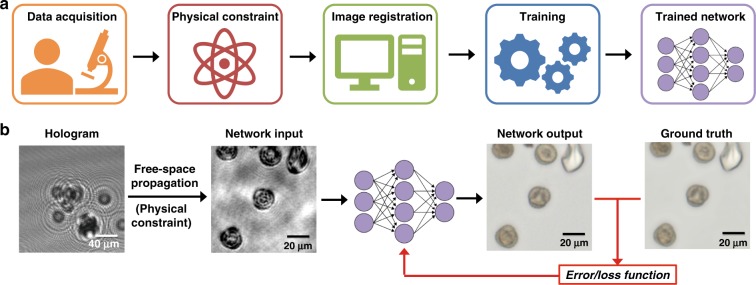
Training a deep neural network for coherent image reconstruction. **a** Training workflow of an image reconstruction deep neural network, including data acquisition, physics-based constraints, image registration, and training. **b** Typical network training and testing procedure, where the network learns to match the input image to a target label (ground-truth image) using a given loss/cost function

Recent developments in the field of deep learning have opened up exciting avenues for significantly advancing holography and coherent imaging systems by circumventing some of these challenges of coherent imaging systems while taking full advantage of their inherent benefits. We believe that this emerging body of exciting work on deep learning in holography will be the key to the wider-scale dissemination and adoption of holographic imaging and sensing systems in the life sciences, biomedicine and engineering fields at large, and it has already been applied to various important tasks in coherent imaging, such as phase recovery^3–6^, super-resolution^7^, phase unwrapping^8,9^ and label-free sensing^10–12^. These methods are generally enabled by the supervised optimization of deep convolutional neural networks (CNNs) using accurately registered image data (Fig. 1a). CNNs typically contain tens to hundreds of layers of convolution kernels (filters), bias terms, and nonlinear activation functions, inspired by biological neural processing. Through the process of *training* (which is a one-time effort), the weights of these filters and biases of the neural network are adjusted in a way that minimizes the error between the network output image and the “gold standard” target labels in terms of a user-defined cost or loss function (for example, mean squared error loss^13^ (Fig. 1b)). This trained network can subsequently be used to perform a predefined image reconstruction task with a single forward-pass through the network, yielding its inference. This reconstruction process typically takes only a fraction of a second (using, e.g., a standard graphics processing unit, GPU) without the need for any iterations, manual tuning of any hyperparameters or refinement of the physical assumptions made regarding the image reconstruction model. In fact, this noniterative single forward-pass reconstruction capability forms one of the major advantages of deep-learning-based solutions to inverse problems in imaging. In the following subsections, we review some of these emerging deep-learning-enabled holographic image reconstruction tasks and exemplify the key opportunities that deep learning brings to the holography and coherent imaging fields.

### Phase recovery and hologram reconstruction

**Fig. 2:**
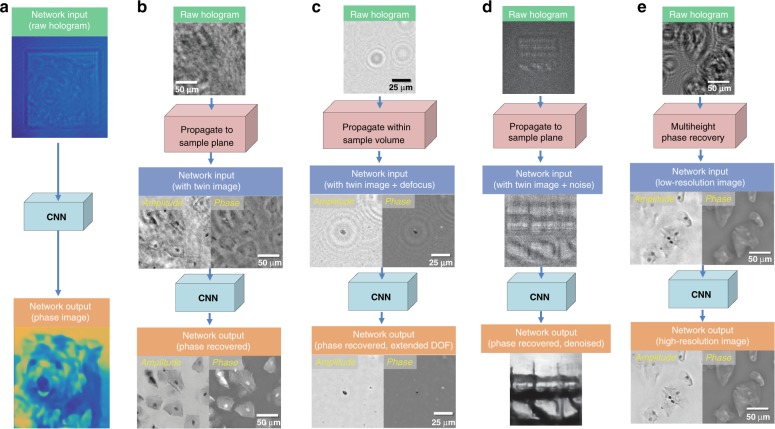
Deep-learning-based hologram reconstruction. **a** An end-to-end CNN was trained to transform a hologram directly to a phase image^4^. Adapted with permission from ref. ^4^. **b** The raw hologram (i.e., without phase information) was numerically focused onto the sample plane and was used as an input for the network to match the phase-recovered image^3^. The sample is a Pap smear specimen. Adapted with permission from ref. ^3^. **c** The raw hologram was propagated to an approximate distance within a sample volume, and the deep network generated an extended depth-of-field reconstruction of the whole sample volume, also performing autofocusing^5^. The specimen is a 3D distributed aerosol sample. Adapted with permission from ref. ^5^. **d** Similar to (**b**) but implemented on holograms under low-photon and poor-SNR conditions^6^. Adapted with permission from ref. ^6^. **e** A CNN was trained to transform a low-resolution holographic reconstruction (created using iterative multiheight phase recovery) to an equivalent high-resolution image of the same sample FOV^7^. The sample is a Pap smear specimen. Adapted with permission from ref. ^7^

One of the most important tasks in holography is phase recovery, as an opto-electronic sensor is only sensitive to the intensity of impinging light. The specifics of the phase recovery process depend on the holographic encoding strategy used, one of which is in-line holography, where the sample wave and the reference wave copropagate in the same direction. As an alternative encoding method, in off-axis holography, there is an angle between the sample and the reference wave directions^14^. In-line holography is generally desirable in various microscopy applications because of its simpler imaging configuration and higher space-bandwidth product (permitting higher resolution over larger fields of view) compared with off-axis holography. For in-line holography, many optical and/or numerical methods have been proposed to retrieve the missing phase information analytically or iteratively^15^, using, e.g., additional hardware to acquire measurements at different axial distances, illumination angles, wavelengths, or polarization states, among other degrees of freedom, where these additional measurements are used as physical constraints for an analytical and/or iterative reconstruction algorithm to converge to a solution. Because multiple measurements are needed for the same object, these systems are generally limited to quasi-static objects. Furthermore, these algorithms are often time-consuming and require tedious tuning of user-defined parameters for convergence to a satisfactory complex-valued image. In contrast to these physics-driven hologram reconstruction approaches, emerging data-driven alternatives based on deep learning have recently demonstrated rapid and robust holographic image reconstruction from a single hologram. These data-driven approaches use accurately registered and labeled image data to train a CNN; these high-quality image labels, used as the ground truth for the training phase, can be obtained from, e.g., known sample structures^4^ (Fig. 2a) or by using a physics-based iterative reconstruction method^3^ (Fig. 2b). After its training, the network can blindly transform a distorted, low-quality image obtained from a single hologram intensity into the desired high-quality label/image^3,4^ (Fig. 2). In general, a better reconstruction quality can be achieved through physics-based learning approaches, for example, by first refocusing the hologram (without phase recovery) onto the object plane and then using deep-learning-based inference (see, e.g., Fig. 2b, d).

It should be noted that the trained deep network can output solutions that deviate from physics-based reconstructions as the network is charged with a certain transformation that cleans twin-image artifacts (defocused replica of the object’s image that overlaps with the real image due to the missing phase information) as well as other interference-related artifacts such as multiple reflection interference or out-of-focus fringes that are present at the network input image (see, e.g., Fig. 2b, c). As an example, a trained network, in its inference, rejected dust particles that were outside the sample plane^3^, although those particles were physical objects imaged in the recorded hologram. Stated differently, the network interpreted these objects as out-of-focus noise, which is a major advantage, as it cleans up the unwanted interference artifacts that are normally present in holographic images due to coherence. As another major advantage, these deep-learning-based hologram reconstruction methods also demonstrate a 4-fold to 30-fold increase in the reconstruction speed compared with the state-of-the-art iterative hologram reconstruction algorithms^5,6^. In fact, these deep-learning-based hologram reconstruction approaches have already been used to empower different computational imaging and sensing devices, such as field-portable and cost-effective imaging flow cytometers^11^, enabling label-free and high-throughput screening of large volumes of liquid samples in field settings.

### Depth-of-field enhancement and autofocusing

A recent work^5^ further demonstrated the ability of a trained deep neural network to perform *simultaneously* autofocusing and phase recovery to generate an extended depth-of-field image from a single hologram measurement. This result could not be obtained by standard iterative hologram reconstruction methods that are based on wave propagation (Fig. 2c). In this deep-learning-based framework, which is termed *Holographic Imaging using Deep Learning for Extended Focus* (HIDEF), the network is trained using pairs of randomly defocused (backpropagated) holograms and their corresponding in-focus, phase-recovered images. HIDEF significantly decreases the time complexity of holographic image reconstruction in 3D through simultaneous refocusing and phase recovery of 3D distributed sample points, which are performed in parallel. HIDEF can be especially useful for wide-field, holographic imaging applications by digitally bringing a large sample volume into focus in real time. For instance, this deep-learning-based reconstruction method was used to characterize particle aggregation-based biosensors over a wide imaging field of view (FOV) greater than 20 mm^2^ and achieved high throughput and the rapid detection of viruses^12^. In another example, a portable and cost-effective device that senses bioaerosols in the field was developed, which used HIDEF to rapidly reconstruct the microscopic images of captured bioaerosols for their automatic detection and label-free classification, achieving an accuracy of >94% for different types of pollen and mold spores^10^.

### Resolution and SNR (signal-to-noise-ratio) enhancement

In addition to the reconstruction of holograms, deep learning has also been used to perform resolution enhancement in coherent imaging systems in two different ways^7^: (1) to surpass the pixel pitch sampling limit in lensless holography systems (Fig. 2e) and (2) to enhance the resolution in diffraction-limited, lens-based holography systems. In the former case, since modern image sensors have a pixel pitch of ~1–2 µm, the resolution of a lensless microscopy system is limited, especially for on-chip holography due to its unit magnification. Conventionally, to surpass this limit, multiple images of the same sample with subpixel shifts are taken to digitally synthesize a high-resolution holographic image, which is known as pixel super-resolution^15^. In ref. ^7^, a neural network was trained to transform lower-resolution images synthesized from fewer numbers (e.g., 1 or 4) of subpixel-shifted measurements to match higher-resolution images synthesized from more (e.g., 36) subpixel shifted measurements, which significantly reduced both the number of hologram measurements and the reconstruction computation time. In the latter case of lens-based coherent imaging, the resolution and FOV are coupled to each other by the space-bandwidth product of the objective lens, where a higher-resolution image can be obtained through a higher magnification and higher NA objective lens at the cost of a smaller FOV. In ref. ^7^, another neural network was trained to transform lower-resolution (lower-NA) holographic reconstructions into higher-NA equivalent complex-valued images, which improved the image resolution beyond the diffraction limit defined by the NA of the objective lens and increased the overall space-bandwidth-product of the lens-based holographic system while also enabling quantitative phase imaging with better resolution and higher throughput (i.e., wider FOV). In both of these cases, through experimental data, with well-controlled imaging conditions and sample sets, the deep neural networks learned to robustly perform frequency extrapolation to infer higher-frequency features without generating artifacts^7^. In addition to in-line holography, another beneficiary of deep-learning-based super resolution in coherent imaging can be off-axis holography, which has a smaller space-bandwidth product in comparison with that of in-line holography while offering a superior overall sensitivity^16^.

Deep learning has also been used to perform denoising of object images reconstructed from their holograms to substantially increase the SNR of the output images^6,17^ (Fig. 2d). In one of these approaches^17^, the network was trained using high-SNR images as the labels, along with computationally simulated input images that had lower SNRs. The trained network was then used to blindly perform robust speckle noise reduction in experimentally obtained image data. A similar framework^6^ was also used to successfully retrieve the shape of an object from its photon-starved hologram with an SNR that is close to one.

### Brightfield holography

One of the landmark attributes of holography is its ability to encode the 3D information of the sample volume using a snapshot 2D interference pattern, that is, the hologram. However, a reconstructed hologram traditionally falls short in terms of its image contrast and axial sectioning capability, which is compromised not only by the twin image, self-interference and speckle artifacts but also by the defocused object features within the sample volume due to the large spatial and temporal coherence. As a result, the digital refocusing of the volumetric sample hologram onto different axial planes results in both in-focus structures and out-of-focus spatial crosstalk from other planes. Overall, the limited contrast generated in holographic reconstruction might be considered one of the significant drawbacks of 3D coherent imaging compared with, for example, the images acquired by a high-NA brightfield scanning microscope.

**Fig. 3:**
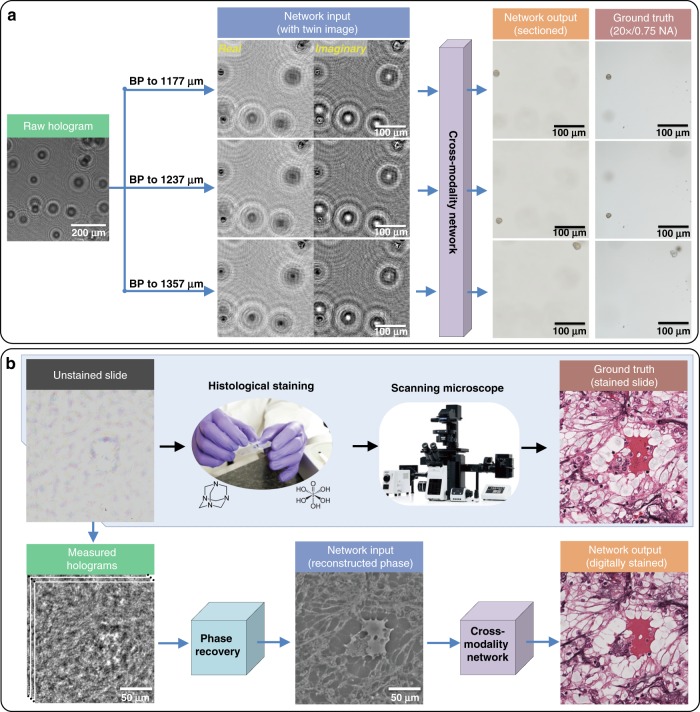
Cross-modality transformations in holography. **a** Brightfield holography^18^. A trained neural network brings the spatial and color contrast and axial sectioning capability of a high-NA brightfield microscope to holography. BP back-propagation. Adapted with permission from ref. ^18^. **b** Phase staining^21^. A trained network takes a reconstructed holographic QPI of an unstained tissue sample as input and then outputs the equivalent image of a histologically stained sample, imaged with a brightfield microscope, virtually achieving staining color and contrast *without* histological staining of the sample. Adapted with permission from ref. ^21^

Recently, deep learning has also been used to close this contrast gap between holographic microscopy and brightfield incoherent microscopy^18^ (Fig. 3b). In this deep-learning-based method, termed “Brightfield Holography”, the neural network learns the *cross-modality image transformation* from numerically propagated complex-valued images of a snapshot hologram intensity to equivalent images at the corresponding axial plane obtained by a high-NA scanning brightfield microscope, matching the image contrast and the axial sectioning capability of the latter. In other words, this brightfield holography method achieves the image quality and the contrast expected from a brightfield microscope *but* without any mechanical volumetric axial scan, taking advantage of the best of both imaging modalities, that is, holography *and* brightfield microscopy. This brightfield holography concept and the underlying cross-modality image transformation framework enabled by image data^19,20^ have the potential to facilitate next-generation high-throughput volumetric imaging systems powered by holography, providing enhanced imaging speed, throughput, contrast and 3D sectioning capability using much simpler imaging hardware. In fact, we highlight in the next subsection another exciting opportunity created by the same cross-modality image transformation framework for the holographic color imaging of label-free (unstained) tissue samples.

### Bringing color to holographic images of unstained samples using cross-modality transformations

Unstained/label-free biological specimens, such as cells and thin tissue sections, exhibit low contrast under a standard brightfield microscope, making it impossible to create a meaningful diagnostic image. To generate a high-contrast image under incoherent illumination, a plethora of labeling/staining techniques have been developed. One of the most well-known and widely used techniques is histochemical staining, which is considered to be the gold standard in histopathology. In comparison to brightfield miroscopy, coherent imaging systems, such as quantitative phase microscopy, provide an efficient contrast mechanism for label-free specimens through the phase information channel. However, this phase contrast mechanism is not compatible with standard diagnostic and sample analysis procedures that rely on more than 150 years of accumulated experience, trained experts/professionals, and image processing algorithms. To address this challenge, a virtual histology staining framework based on cross-modality image transformation has been demonstrated^20,21^. As shown in Fig. 3b, through its training, a deep neural network learns to transform the quantitative phase image (QPI) of a label-free tissue sample imaged using a holographic microscope into an equivalent image of the same tissue section imaged by a brightfield microscope *after* its histochemical staining. As holography offers wide-field and high-throughput QPI over a large FOV (e.g., >20–30 mm^2^ per snapshot), this virtual staining method using the phase contrast generated by the refractive index modulation of label-free tissue samples has the potential to profoundly impact histopathology by eliminating the need for histological staining. This will significantly reduce costs and the amount of expert (histotechnologist) time and chemical reagents required; additionally, this will preserve the tissue section to be reused for further molecular analysis, as needed. Virtual staining of the same tissue sample with multiple types of stains, all in parallel, is also possible with this approach, which is currently impossible to achieve with standard histology methods.

### Imaging through scattering media and diffraction tomography

**Fig. 4:**
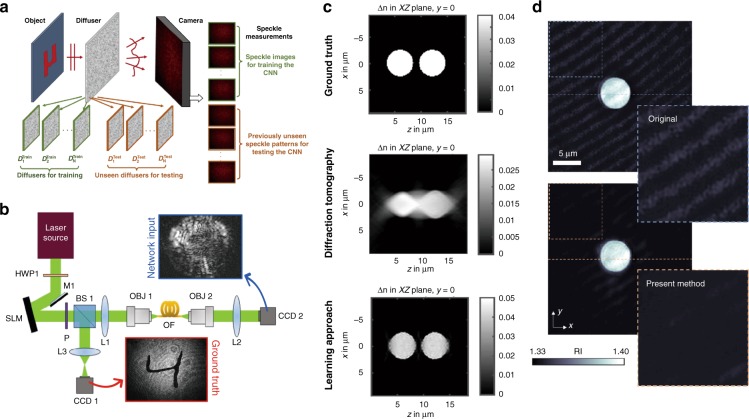
Deep-learning-based imaging through complex media. **a** Imaging through a diffuser^23^. Reprinted with permission from ref. ^23^. **b** Imaging through a multimode fiber^24^. Adapted with permission from ref. ^24^. **c** Learning-based diffraction tomography reconstruction^25^. Reprinted with permission from ref. ^25^. **d** Speckle noise reduction in diffraction tomography^27^. Reprinted with permission from ref. ^27^

The applications of deep learning in coherent imaging systems are not limited to holography, which is based on the assumption of a single-scattering event. Using accurately labeled and cross-registered datasets of input−output image pairs, a deep neural network can also be trained to digitally reverse multiple-scattering events and reconstruct a sample’s image even through scattering media. For example, a deep neural network was successfully trained for image reconstruction through glass diffusers under coherent illumination^22,23^ (Fig. 4a). A related method was also demonstrated to reconstruct and classify handwritten digits from input images of speckle patterns obtained through multimode fiber propagation over a distance of up to 1 km ^24^ (Fig. 4b).

Deep learning has also been applied to optical diffraction tomography. In one of the earlier studies in this field, Kamilov et al. demonstrated that a trained, fully connected neural network can form an inverse model to reconstruct the 3D refractive index distribution of cells from diffraction tomography recordings^25^ (Fig. 4c). Recently, it has also been demonstrated that a CNN can be trained and used for an ill-conditioned inverse imaging problem, providing tomographic reconstruction of densely layered objects from limited angle intensity projection recordings^26^. Another recent work has successfully utilized a generative adversarial network (GAN) for reducing dynamic speckle noise in diffraction tomography images (Fig. 4d), using unregistered pairs of input and label images during the training process^27^.

### Outlook

Deep-learning-based holographic phase recovery, image enhancement/reconstruction, and cross-modality image transformations have profound and broad implications in the field of digital holography and coherent imaging systems, with numerous applications in biomedical imaging, environmental sensing, and materials science, among others. These data-driven image reconstruction methods that are based on deep learning could also be useful for different parts of the electromagnetic spectrum where radiation is harmful to specimens, for example, in X-ray or electron microscopy for probing material properties at the nanoscale. Beyond phase recovery and image reconstruction in a QPI, super-resolution through a deep neural network can further enhance the space-bandwidth product of emerging coherent imaging modalities and might especially be impactful for parts of the spectrum that lack high-pixel-count imagers, such as the infrared (IR) and terahertz (THz) bands. Resolution enhancement could also lead to more extensive uses of holography and coherent imaging in high-resolution and high-throughput microscopy applications, especially those involving portable devices for use in resource-limited settings. In addition, deep-learning-based holography methods bypass the classical trade-off between depth-of-field and axial resolution by enabling high-resolution snapshot volumetric image reconstruction with a significantly extended depth-of-field, which opens up new avenues for high-throughput volumetric imaging applications such as the rapid 3D imaging of cleared organs or tissue samples^28^.

Recent developments such as *brightfield holography*^18^ and *phase staining*^21^ digitally introduce alternative contrast mechanisms to digital holography, which were not possible before the deep-learning-based data-driven approaches were developed. These advances demonstrate the powerful potential of coherent imaging systems that are combined with deep-learning-based statistical image transformations to modify standard image formation, reconstruction and analysis workflows employed in a QPI. We envision that these latest developments will serve as a catalyst to accelerate the translation and wide-scale adoption of holography and coherent imaging techniques in biomedical and clinical applications. Regarding life-science-related applications, live cells can be imaged label-free with low phototoxicity at higher frame rates by using these emerging deep-learning-powered methods and then digitally postprocessed to provide multimodal transformations to other contrast mechanisms for visualization and/or automatic classification or segmentation.

One limitation of the presented deep learning approaches is the need for the creation of accurately registered and matching image datasets to train the networks. However, for various applications, the microscopy field provides an ideal framework for the acquisition of these training image data thanks to the highly repeatable and precise imaging instrumentation and alignment stages that very well control the illumination light properties, sample distances and orientation, which are quite different from, e.g., macroscale imaging under ambient light conditions with traditional camera systems that do not have the same level of control and repeatability as routinely possessed by microscopic imaging systems and instrumentation. This makes the microscopy field a unique and powerful test bed for utilizing the full potential of deep-learning-enabled image reconstructions and transformations at the micro- and nanoscale. Having emphasized this unique advantage of microscopy instrumentation for training neural networks, we also note that a precise registration between the input and label images is not an absolute necessity for deep learning microscopy, and it should be considered as a practical recommendation for the training phase rather than a requirement. In fact, there are various emerging implementations of deep learning toward end-to-end trainable networks that aim to solve inverse problems with no closed-form or iterative solution^29^. In this regard, physical constraints, a priori information or image alignment that feeds into the training image dataset can help us regularize the convergence of a neural network for a given microscopic imaging task and avoid potential hallucinations at the network output (which is especially crucial for biomedical applications). This, however, is not the only means of achieving such competitive generalization performance, and some of the emerging unsupervised learning approaches might bring fundamentally new insights to future uses of deep learning in microscopy.

Despite the advantages brought by the precision of microscopy instrumentation, the transferability of a learned model from one instrument to another remains an issue to be addressed. To use a network model that was trained using one microscopy instrument on a new microscope (which was not part of the training process), the concept of transfer learning^30^ can potentially be used. In this process, a much smaller set of matched input and ground-truth training image pairs can be used to rapidly readjust the formerly trained neural network to work with the new imaging hardware. This process is a one-time effort and can be considered as an initial calibration step for applications of deep learning in microscopy. In fact, during the assembly of each new microscopy instrument, similar calibration and quality control measures are physically implemented to guarantee that each new microscopy instrument performs nominally the same.

In summary, we are experiencing a true renaissance in the holography and coherent imaging fields in general, enabled by a new wave of powerful statistical tools stemming from neural networks and data-driven learning approaches. We believe this is just the beginning of a set of transformative advances that this field will go through, which will not only fundamentally change our imaging instruments and how they work but also open up a plethora of new applications that are not possible with today’s imaging systems.
